# Activity of *Angelica sinensis* extract for cutaneous applications: antioxidant, anti-senescent, and antimicrobial effects

**DOI:** 10.3389/fphar.2026.1779635

**Published:** 2026-03-23

**Authors:** Isabella Giacomini, Veronica Cocetta, Giulia Morandin, Marco Pinzerato, Caterina Dieni, Carolina Frison, Marco Biagi, Paola Brun, Stefano Dall’Acqua, Monica Montopoli

**Affiliations:** 1 Department of Pharmaceutical and Pharmacological Sciences, University of Padova, Padua, Italy; 2 Department of Molecular Medicine, Microbiology Unit, University of Padova, Padua, Italy; 3 Department of Food and Drug, University of Parma, Parma, Italy

**Keywords:** *Angelica sinensis*, anti-inflammatory, antioxidant, anti-senescent, skin health

## Abstract

*Angelica sinensis* (dong quai) is a widely used Traditional Chinese Medicine herb whose constituents are associated with antioxidant and anti-inflammatory activity, making it a plausible candidate for skin-care applications. Here, we evaluated whether an *Angelica sinensis* extract containing 1% ligustilide exerts antioxidant, anti-senescence, antimicrobial, and anti-inflammatory effects in human skin cell models and explored extracellular matrix-related readouts. Human keratinocytes (HaCaT) and dermal fibroblasts (BJ-5ta) were treated with the extract (10, 30, 50 μg/mL); anti-inflammatory activity was assessed by lipopolysaccharide-induced nuclear factor κB (NF-κB) p65 nuclear translocation, antioxidant effects by reactive oxygen species detection with and without hydrogen peroxide challenge, senescence by senescence-associated β-galactosidase after UVB irradiation, and matrix support by collagen type I alpha 1 (COL1A1) immunofluorescence in fibroblasts; scratch-wound assays evaluated migration, and broth microdilution tested activity against *Staphylococcus aureus*, *Staphylococcus epidermidis*, *Cutibacterium acnes*, and *Malassezia globosa*. The extract reduced lipopolysaccharide-induced NF-κB nuclear localization in keratinocytes, lowered basal and hydrogen peroxide-induced reactive oxygen species at 3 and 24 h in both cell types, and attenuated ultraviolet B (UVB)-induced senescence in keratinocyte and fibroblast models; in fibroblasts, it increased COL1A1 signal, while migration was unchanged. The extract showed strong inhibition of *Staphylococcus* species and partial, concentration-dependent inhibition of *C. acnes* and *M*. *globosa* after 24 h, with acceptable viability across the tested range. Overall, these results indicate that *A. sinensis* extract combines anti-inflammatory and antioxidant activity with reduced UVB-associated senescence, fibroblast matrix-supportive signaling, and antimicrobial effects against skin-relevant microbes, supporting further evaluation in more advanced skin models and finished-product formulations before inferring topical performance.

## Graphical Abstract

**Figure:**
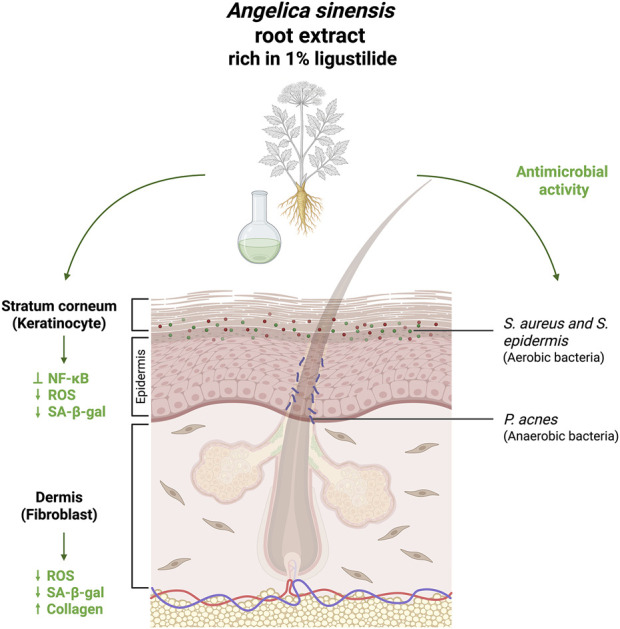
Created in Biorender. Pinzerato, M. (2026) https://BioRender.com/sj5hy6z.

## 1 Introduction

Acute inflammation in the skin is a local protective reaction to injury, microbial invasion, solar irradiation, and environmental pollutants. When environmental or endogenous stressors persist or when epidermal structure and function are altered, this response can become chronic, driving oxidative injury, extracellular-matrix (ECM) remodelling, premature ageing, and carcinogenic change (Agrawal et al., 2023; Pfisterer et al., 2021; Zhang et al., 2024a). Among solar insults, ultraviolet B radiation (UVB, 280–320 nm) generates reactive oxygen species and DNA photolesions that activate pro-inflammatory signaling and a sustained DNA-damage response converging on cellular senescence (Bauwens et al., 2023; Qiang and Dai, 2024). The ensuing senescence-associated secretory phenotype (SASP) sustains low-grade inflammation, perturbs ECM turnover, and compromises dermal repair, thereby linking UVB exposure to photoaging (Salminen et al., 2022). Beyond exogenous stressors, dysbiosis of cutaneous bacteria and fungi can amplify cytokine production and oxidative stress, thereby sustaining inflammation that intersects with senescence pathways (Azzimonti et al., 2023).

Plant-derived, polyphenol-rich extracts are investigated as multimodal modulators of these axes, with reported antioxidant, anti-inflammatory, photoprotective, antibacterial, and wound-healing activities (De Lima Cherubim et al., 2020; Di Salvo et al., 2023; Działo et al., 2016; Farha and n, 2024; Han and Guo, 2012; Hu L. et al., 2025; Yuan et al., 2024). *Angelica sinensis* (Oliv.) (Diels) (dong quai; Radix *Angelicae sinensis*) is a well-characterized herb widely used in traditional East Asian medicine. Consistent with a cutaneous focus, *A. sinensis* (AS) has a documented history of use in traditional Chinese medicine (TCM) in the context of traumatic injuries and inflammatory skin lesions (e.g., sores/boils/carbuncles/ulcers), including topical wound-related preparations (Hsiao et al., 2012; Me et al., 2013; Wagner et al., 2011; Qian et al., 2013). These extracts are rich in phthalides (e.g., ligustilide), phenolic acids (e.g., ferulic acid and other hydroxycinnamic derivatives), and polysaccharides. These constituents have been associated with antioxidant capacity and modulation of inflammatory signaling (e.g., NF-κB/mitogen-activated protein kinase (MAPK)) (Chao and Lin, 2011; Song et al., 2023; Wu et al., 2015).

Despite its broad topical and systemic use, the anti-senescent potential of *A. sinensis* in human cutaneous models remains insufficiently defined, and possible cell-type specificity between epidermal keratinocytes and dermal fibroblasts is poorly characterized.

To address this gap, we evaluate the effects of an extract of *A. sinensis* (AS) on stress-induced senescence in human keratinocytes (HaCaT) and dermal fibroblasts (BJ-5ta), aiming to delineate underlying mechanisms and potential cell-type specificity, and on the viability of bacteria and fungi. We examine the hypothesis that this extract demonstrates antioxidant and anti-inflammatory properties in both human cell types, with potential cell-specific effects on senescence markers and ECM remodeling. Moreover, the extracts of AS reported a significant reduction in the survival of skin-associated microbes, supporting its role in infection-related inflammation. By providing a detailed mechanistic understanding of the effects of *A. sinensis* on skin cells, this work aims to bridge the gap between traditional use and evidence-based application in modern skincare formulations.

## 2 Materials and methods

### 2.1 Angelica extract: source and chemical characterization

An *A. sinensis* (AS) root powder extract (Sergio Fontana s. r.l., Canosa di Puglia (BT), Italy), claimed to contain 1% ligustilide and prepared using Ethanol 40/Water 60 (v/v) as the extraction solvent, was used for all experiments. AS powder was dissolved directly in the complete culture medium; therefore, vehicle controls consisted of the same medium without AS.

For the analysis of the phytochemical constituents, we adopted a comprehensive approach.

Phytochemical analysis was performed using liquid chromatography coupled with diode array detection and multiple-stage fragmentation mass spectrometry (LC-DAD-MS^n^) on an Agilent 1260 Infinity system equipped with an Agilent MS500 Ion Trap mass spectrometer (Agilent Technologies) (Zhang et al., 2009). The instrument operated in both positive and negative electrospray ionization (ESI) modes. General MS parameters included a drying gas pressure of 40 psi, a nebulizer pressure of 25 psi, and a drying gas temperature initially set at 310 °C at time zero, decreasing to 290 °C over 25 min. In negative ion mode, the needle voltage was 4500 V, capillary voltage 85 V, RF loading 85%, and mass spectra were collected over an *m/z* range of 100–1,500.

Chromatographic separation was achieved using an Agilent XDB C18 column (3.0 × 150 mm, 3.5 micron). The mobile phase consisted of water with 1% formic acid (A), acetonitrile (B), and methanol (C). The flow rate was set at 0.400 mL/min throughout the analysis. The gradient program (Supplementary Table S1) was as follows: 0 min, 95% A/0% B/5% C; 15 min, 90% A/5% B/5% C; 30 min, 65% A/10% B/25% C, held isocratic until 35 min; 45 min, 30% A/20% B/50% C; 55 min, 0% A/40% B/60% C; 60 min, 0% A/70% B/30% C; and the system was returned to the initial conditions at 65 min. Detection was performed using a diode array detector, monitoring the spectral range between 200 and 400 nm. For quantification purposes, the following reference compounds were used. Chlorogenic acid, apigenin, hesperidin, and ligustilide (Sigma Aldrich, St. Louis, United States) were used for quantitative analysis. Chlorogenic acid was used to quantify all the caffeoyl quinic acid derivatives. The calibration curve was obtained in the range of concentrations 1–50 ug/mL, and peak areas were collected at 330 nm for chlorogenic acid, 280 nm for apigenin and hesperidin, 254 nm for ligustilide.

For the UPLC-QTOF analysis, an Acquity H-class UPLC coupled with an Xevo QTOF (Waters) operating in electrospray mode was used. The Column was a Waters BEH c18 2.1 × 150 mm (1.7 micron), and the temperature was kept at 65 °C. Gradient uses water, 0.1% formic acid, acetonitrile, and methanol, and the flow rate was 300 μL/min. The gradient program (Supplementary Table S2) was as follows: 0.0–2.0 min, 95% A/5% B (isocratic); 2.0–14.5 min, linear change to 10% A/15% B/75% C; 14.5–15.0 min, 10% A/15% B/75% C (isocratic); 15.0–16.5 min, linear change to 0% A/25% B/75% C; 16.5–17.0 min, 0% A/25% B/75% C (isocratic); then the system was returned to the initial conditions at 18.0 min (re-equilibration). The spectrometer operates with an electrospray voltage of 3.25 V, 700 L/h of drying gas, and a temperature of drying gas of 450 °C. To accurately measure mass, LockMass solution (leucine-enkephalin) was used and sprayed through LockSpray at a flow rate of 20 μL/min. Continuum data were collected in the *m/z* range 50–1,500, and then spectra were centroided, and the *m/z* values were corrected by lock mass *m/z* values.

### 2.2 Cell lines

HaCaT cells (Human Spontaneously Immortalized Keratinocytes) were cultured in High Glucose Dulbecco’s modified Eagle’s medium (DMEM) (Corning, Corning, New York, United States) supplemented with 10% fetal bovine serum (Life-Technologies, Waltham, Massachusetts, United States), 2 mM L-glutamine, 100 U/mL penicillin, and 100 μg/mL streptomycin (Lonza, Basilea, Switzerland). BJ-5ta (Human dermal fibroblasts) cells (ATCC, LGC Standards, Milano, Italy) were cultured in 4:1 mixture of High Glucose Dulbecco’s modified Eagle’s medium (DMEM) and Medium 199 (Corning, Corning, New York, United States) supplemented with 10% fetal bovine serum (Life-Technologies, Waltham, Massachusetts, United States), and 100 U/mL penicillin, and 100 μg/mL streptomycin (Lonza, Basilea, Switzerland). The four parts of DMEM contain 4 mM L-glutamine (Lonza, Basilea, Switzerland), and 1.5 g/L sodium bicarbonate (Sigma Aldrich, St. Louis, United States). Cells were maintained under a humidified atmosphere of 5% CO_2_ in air and incubated at 37 °C.

### 2.3 MTT assay

To determine cell viability, the 3-(4,5-dimethylthiazol-2-yl)-2,5-diphenyltetrazolium bromide (MTT) assay was used (Van Meerloo et al., 2011). HaCaT or BJ-5ta cells (3 × 10^3^ cells/well) were seeded in 96-well plates and, after overnight incubation, treated with AS (10, 30, or 50 μg/mL) in 200 µL complete medium per well; untreated controls received medium only. After 24 h, 20 µL/well of MTT solution (5 mg/mL; Sigma-Aldrich, St. Louis, USA) was added, and plates were incubated for 4 h at 37 °C. The supernatant was then removed, and the formazan crystals were dissolved in 200 µL of acidic isopropanol. Absorbance was measured at 570 nm using a VICTOR Nivo plate reader (Perkin Elmer, Waltham, Massachusetts, United States). Cell viability was reported as % viability relative to the untreated control (set to 100%), calculated as (Abs_sample/Abs_control) × 100. Experiments were performed with n = 3 independent biological replicates, each with four technical replicates per condition.

### 2.4 Immunofluorescence staining

#### 2.4.1 NF-kB

HaCaT (7.5 × 10^4^ cells/well) were seeded in glass coverslips (pre-coated with 0.01% of bovine collagen type I solution (Sigma Aldrich, St. Louis, United States) in 24-well plates in 1 mL of medium. Following overnight incubation, the medium was removed, and cells were pre-treated for 24 h with AS (30, 50 μg/mL), then LPS (Lipopolysaccharide from *Escherichia coli*, serotype O111:B4) stimulation (250 ng/mL) (Sigma Aldrich, St. Louis, United States) was added for 2 h. This protocol was validated in HaCaT cells, starting from literature studies (Lin et al., 2024; Lv et al., 2013). At the timepoint, cells were fixed with 4% paraformaldehyde for 15 min, permeabilized with Triton X-100 0.1% for 5 min, and labelled by incubating with primary antibody for NF-kB p65 (Santa Cruz Biotechnology, Dallas, United States) for 1 h at 37 °C. After PBS wash, cells were incubated with secondary antibody Alexa Fluor 488 anti-rabbit (Invitrogen, Waltham, Massachusetts, United States) for 1 h at 37 °C. Nuclei were stained with 4′,6-diamidino-2-phenylindole (DAPI) (Invitrogen, Waltham, Massachusetts, United States). The coverslips were mounted on glass slides by using Mowiol 40-88 (Sigma Aldrich, St. Louis, United States). Images were acquired through a confocal microscope (Zeiss LSM ×800, ×63 oil magnification). For each independent experiment, one coverslip per condition was analyzed. NF-κB activation was quantified in ImageJ by measuring the p65 fluorescence in nuclear and cytoplasmic ROIs for each cell; cells were classified as p65 nuclear-positive when the nuclear signal predominated over the cytoplasmic signal, and results were reported as the percentage of nuclear-positive cells. Quantification was performed on 20–30 cells per field and five random fields per coverslip per condition; values were normalized to untreated control or LPS-only, as appropriate. Experiments were performed with n = 3 independent biological replicates.

#### 2.4.2 Collagen production

BJ-5ta (3 × 10^4^ cells/well) cells were seeded in glass coverslips in 24-well plates in 1 mL of medium. Following the overnight incubation, the medium was removed, and cells were treated for 24 h with AS 30 μg/mL. Then, cells were fixed with 4% paraformaldehyde for 15 min, permeabilized with Triton X-100 0.1% for 5 min, and labelled by incubating with primary antibody for COL1A1 (Cell Signaling Technology, Danvers, Massachusetts, United States) for 1 h at 37 °C. After PBS wash, cells were incubated with secondary antibody Alexa Fluor 488 anti-rabbit (Invitrogen, Waltham, Massachusetts, United States) for 1 h at 37 °C. Nuclei were stained with Hoechst 33342 (Invitrogen, Waltham, Massachusetts, United States). The coverslips were mounted on glass slides by using Mowiol 40-88 (Sigma Aldrich, St. Louis, United States). Images were acquired through a confocal microscope (Zeiss LSM ×800, ×63 oil magnification). For each independent experiment, one coverslip per condition was analyzed. COL1A1 signal was quantified in ImageJ as mean fluorescence intensity per cell (COL1A1 channel intensity normalized to nuclei count) and expressed relative to untreated control (set to 1), analyzing 20–30 cells per field and five random fields per coverslip per condition. Experiments were performed with n = 3 independent biological replicates. Ascorbic acid 100 µM was used as a positive control (n = 2).

### 2.5 Reactive oxygen species (ROS) production

3 × 10^3^ HaCaT and BJ-5ta cells/well were seeded in 200 μL of medium in 96-well black plates and incubated overnight at 37 °C. The next day, the culture medium was aspirated and replaced with fresh complete medium (200 μL) containing AS (10, 30, 50 μg/mL). Both HaCaT and BJ-5ta cells were treated for 3 or 24 h in separate experiments. After the treatment, the medium was removed and cells were incubated with diacetylated 2′,7′-dichlorofluorescein (DCF-DA) probe (Merck, Darmstadt, Germany) at 100 μM (HaCaT) or 50 μM (BJ-5ta) for 30 min at 37 °C. Then, the basal fluorescence was measured using a VICTOR Nivo plate reader (Ex 485 nm, Em 535 nm). Immediately after the basal reading, H_2_O_2_ was added directly to the wells (final concentration: 5 mM for HaCaT or 2.5 mM for BJ-5ta) to induce acute oxidative stress, and fluorescence was measured again. This assay takes advantage of the fluorescence emitted by the oxidation of the non-fluorescent DCF-DA and evaluates the intracellular ROS production. N-acetyl-L-cysteine (NAC) was used as a positive control. Data were expressed as relative fluorescence units (RFU) and normalized to the basal untreated control, set as 1. Controls included untreated cells and H_2_O_2_-only challenged cells. Experiments were performed with n = 3 independent biological replicates, each with four technical replicates per condition.

### 2.6 Scratch-wound assay

The scratch-wound assay was used to evaluate *in vitro* cellular migration. HaCaT and BJ-5ta cells were seeded in 6-well plates, reached 100% confluent monolayers, and then scratched with a sterile 200-µL pipette tip, rinsed to remove debris, and incubated in 3 mL of medium with AS 30 μg/mL or with medium only. Phase-contrast images were taken at 0, 3, 6, 9, and 24 h (Nikon Ti-S, 10×; Nexcope NIB620-FL, 4×). Wound area was quantified with ImageJ software and expressed as closure relative to t_0_ (Arvia et al., 2024; Pressi et al., 2023), which was calculated as (Area_t_0_ − Area_t)/Area_t_0_. For each experiment, two wounds per condition were analyzed; experiments were independently repeated n = 3 times.

### 2.7 Senescence induction

HaCaT or BJ-5ta cells (5 × 10^5^) were seeded in T75 flasks (75 cm^2^) (Sarstedt, Nümbrecht, Germany) in 10 mL complete medium. After overnight incubation, cells were treated with AS 30 μg/mL for 24 h before UVB exposure. Then, the medium was removed, and cells were covered with PBS during irradiation. Cells were irradiated with UVB to a final dose of 220 mJ/cm^2^ using an irradiation chamber (Opsytec Dr. Gröbel GmbH, Ettlingen, Germany), with exposure automatically controlled by the instrument to deliver the preset dose. Immediately after irradiation, PBS was replaced with fresh complete medium containing AS 30 μg/mL, while control cells received medium only. In parallel, non-irradiated growth controls (±AS) were processed identically but not exposed to UVB. Cells were cultured for 5 days to develop a senescent phenotype and then used for further experiments (Zumerle et al., 2024). The 30 μg/mL concentration was selected as the minimum effective concentration identified in ROS screening while maintaining acceptable viability.

#### 2.7.1 Senescence-associated β-galactosidase assay (SA-β-gal assay)

At day 5 post UVB irradiation, senescence was assessed using the SA-β-gal staining kit (Cell Signaling Technology, Danvers, Massachusetts, United States) following the manufacturer’s instructions. Senescence-associated β-galactosidase (SA-β-gal) staining (pH 6.0) was used as a widely adopted readout of stress-induced senescence and was interpreted alongside complementary molecular markers (e.g., CDKN1A/p21) (Zumerle et al., 2024; Itahana et al., 2013; Dimri et al., 1995; Ajoolabady et al., 2025; Ogrodnik et al., 2024). Quantifications were conducted on at least six images per experiment by calculating the ratio of perinuclear positive blue cells to perinuclear blue negative cells. Fluorescent nuclear staining was carried out using Hoechst 33342 (ThermoFisher Scientific, Waltham, Massachusetts, United States). All images were acquired with Nexcope NIB620-FL (Bresser, Rhede, Germany) and then analyzed with ImageJ software. For each condition, at least five fields were quantified per experiment; experiments were independently repeated n = 5 times.

#### 2.7.2 RT-qPCR

Gene expression was quantified by reverse transcription quantitative PCR (RT-qPCR). At day 5 post UVB irradiation, total RNA was extracted from the samples using the Direct-zol RNA MiniPrep® kit (Zymo Research, Irvine, California, United States). Complementary DNA (cDNA) synthesis was performed using the Reliance Select cDNA Synthesis Kit (Bio-Rad, Hercules, California, United States). Quantitative real-time PCR (qPCR) reactions were carried out with SsoAdvanced Universal SYBR® Green Supermix (Bio-Rad, Hercules, California, United States) on a CFX Duet Real-Time PCR System (Bio-Rad, Hercules, California, United States) using Bio-Rad CFX Maestro software. The threshold cycle (Ct) for *CDKN1A (P21)* (Fw: 5′-TGT​CCG​TCA​GAA​CCC​ATG​C-3’; Rv: 5′- AAA​GTC​GAA​GTT​CCA​TCG​CTC-3′) was determined automatically and normalized to the geometric mean of the housekeeping gene *β-ACTIN* (Fw: 5′-CCA​ACC​GCG​AGA​AGA​TGA-3′; Rv: 5′-CCA​GAG​GCG​TAC​AGG​GAT​AG-3′) (ΔCt value). The relative gene expression levels were calculated using the 2^−ΔΔCt^ method (Livak method).

### 2.8 Antimicrobial assay

The antimicrobial activity was evaluated against microbes involved in skin infection and acne. Specifically, *Staphylococcus aureus* (strain designation, NCTC 8532) and *Staphylococcus epidermidis* (strain designation, NCTC11047) were cultured in Trypticase soy broth or agar (Thermo Fisher Scientific, Waltham, Massachusetts, United States) at 37 °C. *Cutibacterium acnes* (strain designation, NCTC737) was grown in Modified Reinforced Clostridial broth (ATCC Medium 2107, Manassas, Virginia, United States) or Trypticase soy agar added with defibrinated sheep blood (ATCC Medium 260, Manassas, Virginia, United States) at 37 °C under anaerobic conditions. *Malassezia globosa* (strain designation, CBS 7966) was cultured in Sabouraud’s dextrose broth or agar at 30 °C. Microbial strains were purchased from ATCC (LGC Standards; Milan, Italy). Antimicrobial activity of AS was assessed using the microplate dilution method as already described, with minor modifications (Ray et al., 2013). Microbes were grown in the appropriate culture broths, centrifuged, and diluted in fresh broth to obtain an optical density (OD) 0.5 McFarland corresponding to 1.5 × 10^8^ colony-forming units (CFU)/mL. Microbial suspensions (100 μL) were dispensed into 96-well plates. AS was added as a concentrated working solution (2 µL per well), and wells were brought to a final volume of 200 µL/well with the corresponding growth medium, yielding final AS concentrations of 10, 30, and 50 μg/mL. Plates were incubated under the appropriate growth conditions. The growth of bacteria and fungi was assessed after 24 h by recording the OD at 620 nm using the MultiPlateReader VictorX2 (Perkin Elmer). The OD determined in microbes cultured with the proper growth medium only was used as a positive control, with growth arbitrarily set at 100%. AS incubated in culture medium alone (without microbial inoculum) served as a negative control, as it did not result in microbial growth. The effects of AS treatment on microbial growth were calculated by comparison with the untreated controls and reported as % survival. Each condition was tested in three technical replicates within each independent experiment (n = 3 biological replicates).

### 2.9 Statistical analyses

The statistical analyses were assessed by GraphPad Prism software package version 10.2.2. (GraphPad Software, San Diego, California, United States). Group comparisons were analyzed by one-way ANOVA with Dunnett/Tukey *post hoc* tests as appropriate; two-sided t-tests were reserved for single pairwise comparisons. Significance was considered at *p* < 0.05.

## 3 Results

### 3.1 Phytochemical composition

LC-DAD-MS^n^ analysis of the Angelica extract revealed multiple UV-absorbing peaks in the DAD chromatograms (Supplementary Figure S1). A major peak showed an absorption maximum at 330 nm, consistent with hydroxycinnamic acid derivatives. A second peak displayed a UV maximum at 280 nm, attributable to a dihydrophthalide derivative, whereas a third peak exhibited a UV maximum at 280 nm together with a shoulder at 330 nm, suggesting the presence of a flavanone derivative. In addition, a series of later-eluting, more lipophilic constituents was observed between 25 and 30 min, showing marked UV absorbance and tentatively attributable to lipid-derived compounds.

Compound identification was performed by integrating DAD UV spectra with LC-DAD-MS^n^ low-resolution fragmentation and high-resolution accurate-mass measurements acquired on a QTOF instrument operating in MS^e^ mode, enabling assignment of precursor and fragment *m/z* values.

**FIGURE 1:**
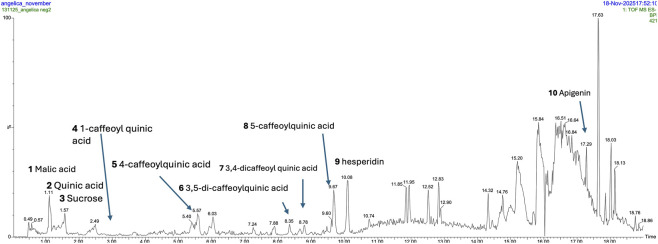
The main compounds identified in the negative ion mode are highlighted in the QTOF chromatogram. UPLC-QTOF chromatogram acquired in negative ion mode, highlighting the main compounds detected in the AS extract. Peak numbers correspond to the compound list reported in Table 1.

In negative ion mode, malic acid and quinic acid were confirmed by injection of reference standards. Several peaks showed UV features consistent with hydroxycinnamic derivatives and shared a pseudomolecular ion [M − H]^−^ at *m/z* 353; accurate-mass data supported the molecular formula C_16_H_18_O_9_ for these caffeoylquinic acid isomers. Two additional peaks exhibited [M − H]^−^ at *m/z* 515, corresponding to C_25_H_24_O_12_, consistent with dicaffeoylquinic acids. Isomer assignment (1-, 3-, 4-, and 5-caffeoylquinic acids; and 3,4-/3,5-dicaffeoylquinic acids) was performed based on diagnostic fragmentation patterns following Clifford et al., 2005 (Clifford et al., 2005), as reported in Table 1. Hesperidin was assigned from accurate-mass measurements (C_28_H_34_O_15_) and characteristic fragments (aglycone) and confirmed by reference standard injection; apigenin was similarly identified by accurate mass (C_15_H_10_O_5_) and confirmed by standard injection. A summary of the species detected in negative mode is shown in Figure 1.

**TABLE 1:** Identified compounds in the Angelica extract, detected in negative ion mode.

Peak number	tr LC	tr UPLC	HR-MS (QTOF) [M − H]^−^	Formula	Fragments	Identification
1	2.05	1.11	133.0139	C_4_H_6_O_5_	​	Malic acid*
2	1.92	1.23	191.0558	C_7_H_12_O_6_	​	Quinic acid*
3	1.98	1.57	341.1071	C_12_H_22_O_11_	​	Sucrose*
4	8.12	3.23	353.0765	C_16_H_18_O_9_	191.0554-179.1952-135.2200	1-caffeoyl quinic acid
5	10.5	5.73	353.0821	C_16_H_18_O_9_	191.0556-179.1952	4-caffeoyl quinic acid*
6	12.4	8.35	515.1193	C_25_H_24_O_12_	353.0821-191.0554	3,5-dicaffeoyl quinic acid*
7	12.6	8.78	515.1193	C_25_H_24_O_12_	353.0821-191.0554	3,4-dicaffeoyl quinic acid
8	13.2	9.67	353.0815	C_16_H_18_O_9_	191.0556-179.1952-135.2200	5-caffeoyl quinic acid
9	14.2	10.1	609.181	C_28_H_34_O_15_	301.0707	Hesperidin*
10	27.2	17.29	269.0462	C_15_H_10_O_5_	227.0379-151.0231	Apigenin*

Compounds are listed, indicating retention time in the LC-DAD-MS^n^ (tr LC), retention time in the UPLC-QTOF (tr UPLC), observed pseudomolecular ion [M − H]^−^ detected in the LC-DAD-MS^n^, and High-resolution *m/z* value of the [M − H]^−^ specie observed in the UPLC-QTOF, principal fragments. Compounds indicated with “*” were confirmed by injecting authentic standards.

Positive ion mode enabled the identification of multiple constituents (Table 2). Peaks with UV spectra compatible with phthalide derivatives showed dominant ions at *m/z* 191, consistent with [M + H]^+^ of E- and Z-ligustilide, a well-established marker compound in Angelica roots (Lu et al., 2004; Zschocke et al., 2005). In addition, several peaks sharing the same nominal mass and consistent accurate-mass formula C_24_H_28_O_4_ were tentatively assigned to isomeric phthalide dimers (angesilides) previously reported from Angelica roots (Alkan Türkuçar et al., 2021; Hu C. et al., 2025; Wen et al., 2025). Minor coumarins (e.g., scopoletin, angelicin, psoralen, and phelloptorin) were also detected at low abundance (Table 2) (Alloush et al., 2022).

**TABLE 2:** Identified compounds in the Angelica extract, detected in positive ion mode.

tr LC	tr UPLC	HR-MS (QTOF) [M + H]^+^	Formula	Fragments	Identification
24.6	12.6	193.0505-215.0325 (+Na^+^)	C_10_H_8_O_4_	137.2243	Scopoletin*
26.5	13.17	191.1078	C_12_H_14_O_2_	173.1269-145.1656	E-ligustilide
26.8	13.42	191.1078	C_12_H_14_O_2_	173.1269-145.1656	Z-ligustilide
27.9	14.86	403.1885 (+Na^+^)-381.2066	C_24_H_28_O_4_	213.2123-191.2433-173.2417	Angeliside A
28.2	14.92	403.1885 (+Na^+^)--381.2067	C_24_H_28_O_4_	213.2123-191.2433-173.2418	Angeliside B
28.2	15.1	403.1885 (+Na^+^)--381.2065	C_24_H_28_O_4_	213.2123-191.2433-173.2419	Angeliside C
28.2	15.52	403.1885 (+Na^+^)--381.2065	C_24_H_28_O_4_	213.2123-191.2433-173.2420	Angeliside D
28.2	15.58	403.1885 (+Na^+^)--381.2066	C_24_H_28_O_4_	213.2123-191.2433-173.2421	Angeliside E
28.2	15.73	403.1885 (+Na^+^)--381.2065	C_24_H_28_O_4_	213.2123-191.2433-173.2422	Angeliside F
28.2	15.86	403.1885 (+Na^+^)--381.2065	C_24_H_28_O_4_	213.2123-191.2433-173.2423	Angeliside G/I
24.5	10.59	187.2329	C_11_H_6_O_3_	159.2212-131.2069-115.2140	Psoralen*
25.1	14.48	187.2308	C_11_H_6_O_3_	131.2069-115.2140	Angelicin*
26.6	15.19	301.1076	C_17_H_16_O_5_	​	Phelloptorin
23.5	11.65	245.1171	C_15_H_16_O_3_	​	Suberosin
27.7	15.83	357.09445	C_17_H_18_O_7_	​	Isobyakangelicin
23.6	9.67	247.06061	C_13_H_10_O_5_	177.0899	Isopimpinellin

Compounds are listed, indicating retention time in the LC-DAD-MS^n^ (tr LC), retention time in the UPLC-QTOF (tr UPLC), observed pseudomolecular ions [M + H]^+^ or [M + Na]^+^ detected in the LC-DAD-MS^n^, and High-resolution *m/z* value of the [M + H]^+^ specie observed in the UPLC-QTOF, principal fragments. Compounds indicated with “*” were confirmed by injecting authentic standards.

Overall, the extract contained several diagnostic marker compounds for Angelica roots. Total ligustilide (E + Z) accounted for 1.2% (w/w) on a dry-weight basis. The cumulative hydroxycinnamic acid derivatives represented 0.15% (w/w), while flavonoids were present at 0.10% (w/w). Coumarins, known minor constituents of *A. sinensis* roots, were detected at low levels (0.12% (w/w)).

### 3.2 Cytotoxicity

**FIGURE 2:**
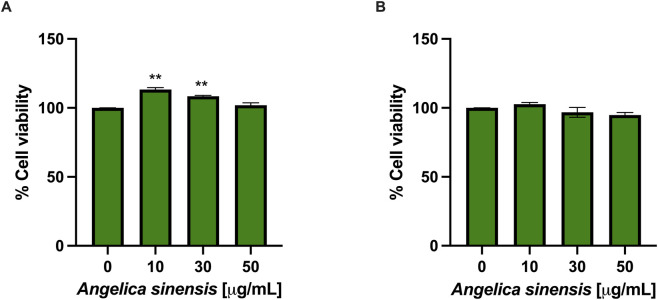
AS does not decrease viability in HaCaT keratinocytes and BJ-5ta fibroblasts after 24 h. **(A)** HaCaT and **(B)** BJ-5ta cells were treated with AS (10, 30, 50 μg/mL) for 24 h, and cell viability was assessed by the MTT assay. Data are shown as % viability relative to untreated control (set to 100%). Data are mean ± SD of n = 3 independent biological replicates. Statistical analysis: one-way ANOVA with Dunnett’s *post hoc* test vs*.* control. **p < 0.01 vs*.* untreated control.

To assess the safety profile of AS, an MTT assay was performed. HaCaT and BJ-5ta cells were treated for 24 h with the extract (10, 30, 50 μg/mL). As shown in Figure 2, AS did not alter the cell viability in both keratinocytes and human fibroblasts.

### 3.3 NF- κB translocation

NF-κB is a sequence-specific transcription factor composed of a family of interconnected transcription factors that bind specific DNA sequences called κB sites and regulate the expression of certain genes. It is involved in modulating metabolic and inflammatory responses, regulating functions of both the innate and adaptive immune systems. In response to a stimulus, NF-κB is activated, and the IκB unit is degraded, allowing NF-κB to translocate into the nucleus and leading to increased expression of pro-inflammatory genes. AS has a wide range of pharmacological activities, including immunomodulation, antioxidant, anti-inflammatory, and anti-fibrosis (Ren et al., 2025). Moreover, its anti-inflammatory activity is already known *in vivo* and confirmed on HaCaT cells (Sun et al., 2024; Zhang et al., 2024b).

**FIGURE 3:**
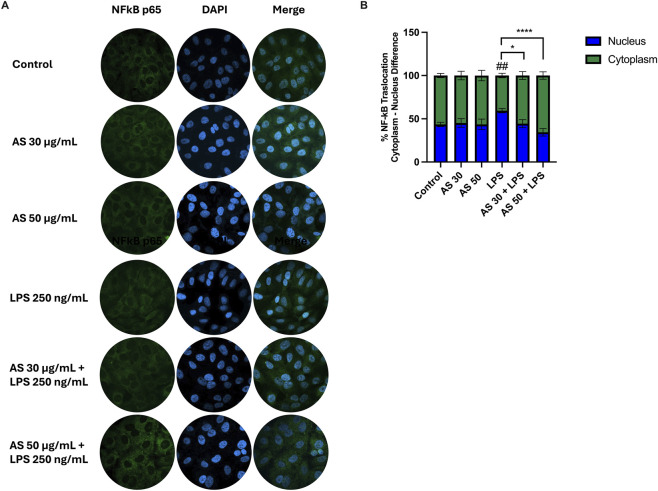
AS inhibits LPS-induced NF-κB activation in HaCaT keratinocytes. **(A)** Representative immunofluorescence images of NF-κB p65. Cells were pre-treated with AS (30, 50 μg/mL) and stimulated with LPS (250 ng/mL, 2 h). Nuclei were stained with DAPI (blue), and NF-κB p65 was detected with Alexa Fluor 488 (AF488) (green). **(B)** Quantification of NF-κB activation expressed as percentage of p65 nuclear-positive cells. Data are mean ± SD from n = 3 independent biological replicates. Statistical analysis: one-way ANOVA followed by Tukey’s *post hoc* vs*.* control or LPS. ^##^ *p* < 0.01, LPS vs*.* control; ^*^ *p* < 0.05, ^****^ *p* < 0.0001, treatment vs*.* LPS.

NF-κB localization was demonstrated by immunocytochemistry using an anti-p65 antibody (green) and DAPI (blue) to label nuclei (Figure 3A). The HaCaT cell line was treated for 24 h with AS extract at 30 and 50 μg/mL and then exposed for 2 h to LPS at 250 ng/mL to mimic an inflammatory state.

Under basal conditions, NF-κB remained predominantly cytoplasmic, with fluorescence levels comparable to those of the control. LPS stimulus increased nuclear localization of NF-κB, whereas pre-treatment with AS significantly attenuated this response at both concentrations, with the greatest effect at 50 μg/mL (reduction in the percentage of p65 nuclear-positive cells by ∼30% vs*.* LPS alone) (Figure 3B).

### 3.4 ROS production

**FIGURE 4:**
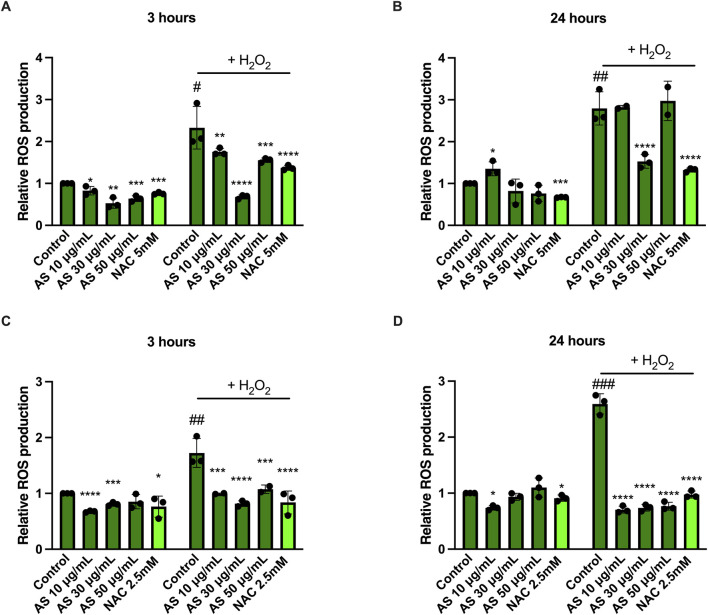
AS modulates intracellular ROS under basal and H_2_O_2_-challenged conditions. HaCaT **(A,B)** and BJ-5ta **(C,D)** cells were treated with AS (10, 30, 50 μg/mL) for 3 h **(A,C)** or 24 h **(B,D)**. ROS was measured by DCF-DA fluorescence under basal conditions (left panels) and following oxidative challenge with H_2_O_2_ (right panels). Data are expressed as RFU normalized to the basal control or H_2_O_2_. Data are mean ± SD from n = 3 independent biological replicates. Statistical analysis: one-way ANOVA with Dunnett’s *post hoc* test vs*.* control. ^*^ *p* < 0.05, ^**^ *p* < 0.01, ^***^ *p* < 0.001, ^****^ *p* < 0.0001, treatment vs*.* control; ^#^ *p* < 0.05, ^##^ *p* < 0.01, ^###^ *p* < 0.001, H_2_O_2_ vs*.* basal control.

Since ROS production is strictly related to skin inflammation and pathogenesis (Chelombitko, 2018), we confirmed the antioxidant activity of AS extract in both cell lines (Figure 4), under basal and stress-induced conditions (H_2_O_2_ increased ROS levels of ∼200% in HaCaT cells and ∼150% in BJ-5ta). In HaCaT cells, AS treatment (10, 30, 50 μg/mL) for 3 h (Figure 4A) reduced ROS levels and attenuated the H_2_O_2_-induced ROS increase. AS 30 μg/mL reduced ROS levels by ∼50% after 3 h, and this reduction was maintained also upon H_2_O_2_ stimulation. After 24 h of treatment (Figure 4B), the antioxidant effect in HaCaT cells was observed, reaching statistical significance only at specific AS concentrations (as shown in Figure 4B), and AS also counteracted the H_2_O_2_-induced ROS increase. In BJ-5ta cells, AS reduced ROS levels under both basal and stress-induced conditions (Figures 4C,D); however, under basal conditions at 24 h, AS 30 μg/mL did not produce a statistically significant decrease in ROS, as shown in Figure 4D.

### 3.5 Scratch-wound assay and collagen production

**FIGURE 5:**
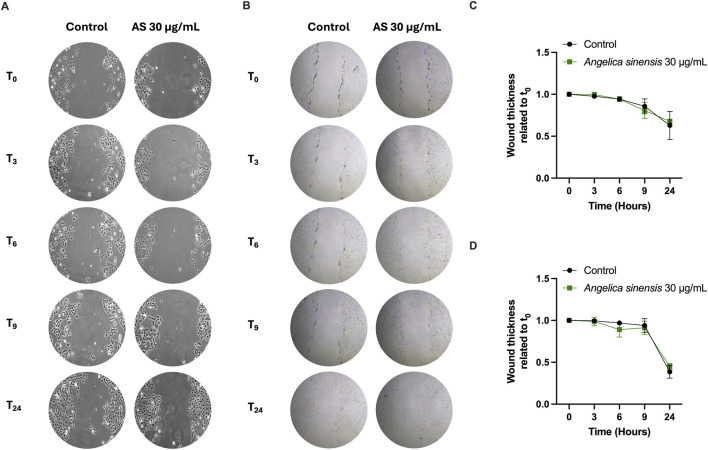
Scratch-wound closure in keratinocytes and fibroblasts following AS treatment. **(A,B)** Representative scratch images in HaCaT keratinocytes **(A;** 10x**)** and BJ-5ta fibroblasts **(B;** 4x**)** treated with AS (30 μg/mL). **(C,D)** Quantification of scratch closure in HaCaT **(C)** and BJ-5ta **(D)**. Wound closure was calculated as closure = (Area_t0 − Area_t)/Area_t0. Data are mean ± SD from n = 3 independent biological replicates. Statistical analysis: unpaired two-tailed Student’s t-test vs*.* control.

Keratinocytes and fibroblasts are implicated in the skin repair process (Wojtowicz et al., 2014). Thus, in this work, the effect of AS on cell migration was assessed. The *in vitro* scratch-wound assay was performed at different time-points (0–3–6–9–24 h) with AS 30 μg/mL. Because the study was designed to focus on the minimum effective concentration, the 50 μg/mL condition was not tested. As shown in Figure 5, AS does not accelerate the repair process of HaCaT (Figures 5A–C) and BJ-5ta (Figures 5B–D) cells with respect to the untreated control.

**FIGURE 6:**
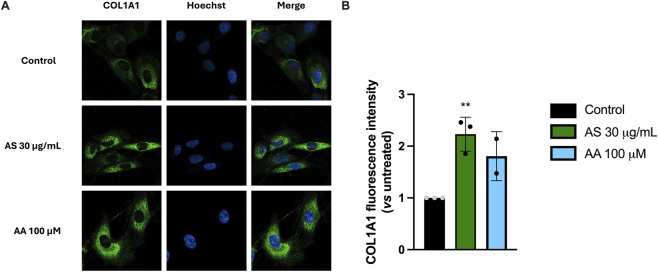
AS increases COL1A1 signal in BJ-5ta fibroblasts. BJ-5ta cells were treated for 24 h with AS (30 μg/mL) or ascorbic acid (AA; 100 µM) as a positive control. **(A)** Representative immunofluorescence images stained for COL1A1 (green) and nuclei (Hoechst, blue). **(B)** Quantification of COL1A1 fluorescence expressed as mean fluorescence intensity per cell (normalized to nuclei count) and reported relative to untreated control. Data are mean ± SD from n = 2 (for AA) or 3 (for AS) independent biological replicates. Statistical analysis: unpaired two-tailed Student’s t-test vs*.* control. ^**^ *p* < 0.01, treated vs*.* control.

We next investigated whether AS extract was able to increase the collagen production in human fibroblasts. As it is presented in Figure 6, AS 30 μg/mL significantly enhances COL1A1 fluorescence intensity (∼220%) compared to the control. Ascorbic acid 100 μM was used as a positive control. The observed rise in collagen production is compatible with a pro-matrix remodeling profile, favoring deposition over turnover within the dermal compartment (Fisher et al., 2023).

### 3.6 Senescence

**FIGURE 7:**
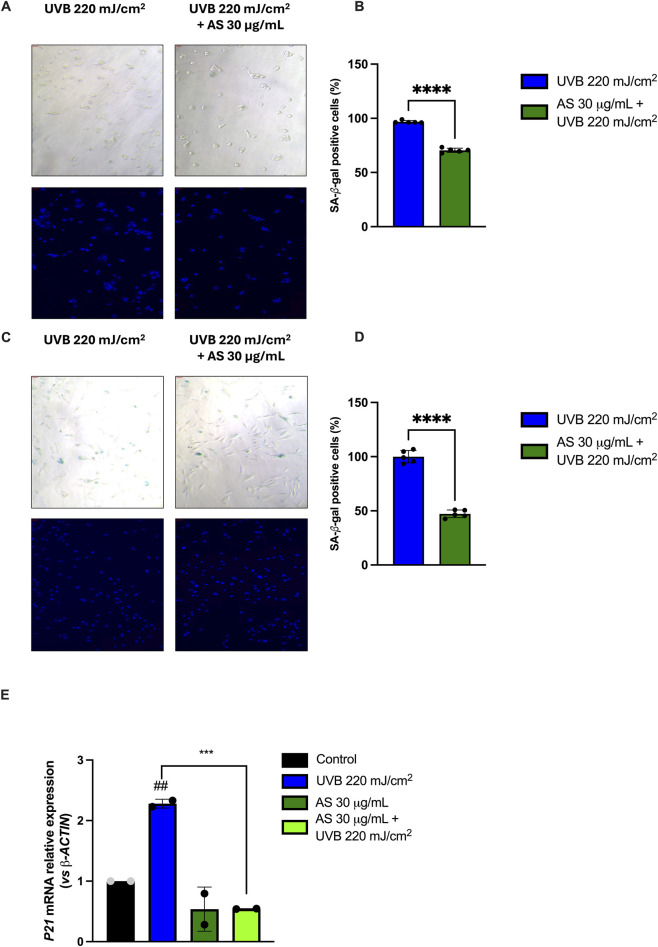
AS reduces UVB-induced senescence markers in keratinocytes and fibroblasts. **(A–C)** Representative SA-β-gal staining images (bright-field, top) with nuclear counterstain (Hoechst, bottom) in HaCaT **(A)** and BJ-5ta **(C)** cells under basal conditions, UVB, and UVB + AS (30 μg/mL). **(B,D)** Quantification of SA-β-gal–positive cells expressed as % of total cells per field in HaCaT **(B)** and BJ-5ta **(D)** Data are mean ± SD of n = 5 independent biological replicates. **(E)** *CDKN1A (p21)* mRNA expression measured by RT-qPCR and reported as relative expression (2^−ΔΔCt^) normalized to β-actin in BJ-5ta cells. Data are mean ± SD from n = 2 independent biological replicates. Statistical analysis: one-way ANOVA with Dunnett’s *post hoc* test vs*.* control. ^***^ *p* < 0.001, ^****^ *p* < 0.0001, treatment vs*.* UVB; ^##^ *p* < 0.01 UVB vs*.* basal control.

Increased levels of ROS have a detrimental effect on cellular integrity and homeostasis, leading to chronic inflammation and accelerating the severity of age-related disorders such as skin aging (Davalli et al., 2016). In light of this evidence, and considering the significant antioxidant activity demonstrated by AS, we next investigated whether this extract could also show anti-senescent properties in both HaCaT and BJ-5ta cells (Figures 7A–C). UVB 220 mJ/cm^2^ induced senescence in both HaCaT and BJ-5ta cells, as it is possible to observe by the significant increase of SA-β-gal positivity (Supplementary Figure S2C–F). Pre-treatment for 24 h with AS 30 μg/mL was able to significantly reduce UVB-induced senescence on HaCaT (∼30%) (Figures 7A,B) and BJ-5ta cells (∼50%) (Figures 7C,D). Pre-treatment in basal condition with AS extract did not alter the SA-β-gal positivity, as shown in Supplementary Figure S2A,B,D,E. To complement SA-β-gal staining, *CDKN1A (p21)* mRNA levels were quantified by RT-qPCR in BJ-5ta cells, showing a significant reduction in its expression (Figure 7E), and supporting an anti-senescent profile of AS.

### 3.7 Antimicrobial activity

**FIGURE 8:**
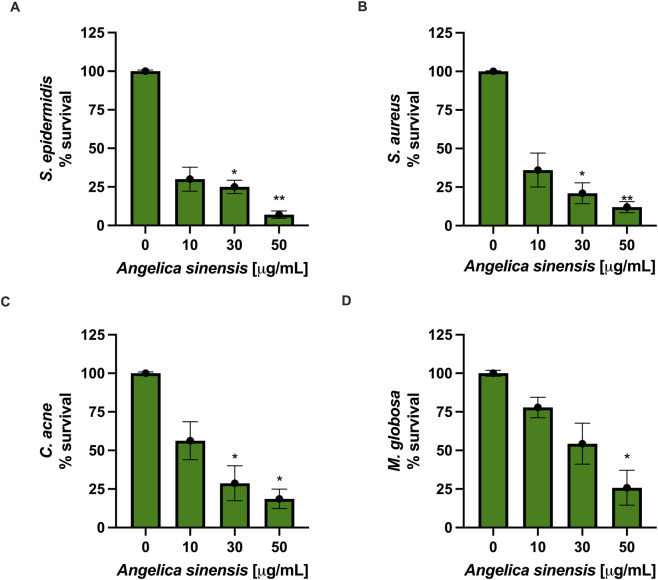
*In vitro* antimicrobial activity of AS against skin-relevant microorganisms. Bacterial **(A–C)** and fungal **(D)** suspensions (1.5 × 10^8^ CFU/mL) were incubated for 24 h with AS (10, 30, 50 μg/mL). Microbial growth was assessed by measuring optical density (OD) and is reported as % survival relative to the no-AS control (set to 100%). Data are mean ± SEM from n = 3 independent biological replicates, each performed in technical triplicate. Statistical analysis: one-way ANOVA with Dunnett’s vs*.* control. ^*^ *p* < 0.05, ^**^ *p* < 0.01, treated vs*.* control.

Incubation with AS affected microbial survival in a concentration-dependent manner. *Staphylococcus* spp. showed the highest sensitivity, with survival almost completely suppressed at 50 μg/mL and markedly reduced also at 10 μg/mL (Figures 8A,B). However, *S. epidermidis* exhibited a slightly greater reduction in viability than *S. aureus* at the highest dose tested. *C. acnes* and *M. globosa* were moderately affected, requiring higher concentrations to observe substantial inhibition and displaying partial growth inhibition at 50 μg/mL (Figures 8C,D). Indeed, at this AS concentration, a residual survival was still detectable. Overall, these findings indicate that AS exerts antimicrobial activity against the most important microbial species involved in acne, skin infection, and inflammation.

## 4 Discussion

Herbal products continue to attract sustained interest in skin care due to their antioxidant and anti-inflammatory properties, which are central to skin homeostasis. Polyphenols are especially relevant: they scavenge reactive species, modulate NF-κB/MAPK signaling, and can reduce downstream matrix damage (De Lima Cherubim et al., 2020; Di Salvo et al., 2023; Farha and n, 2024; Hu L. et al., 2025). To obtain consistent performance, however, plant inputs must be well controlled. Clear botanical identity, appropriate harvest and post-harvest handling, as well as extract standardization, are crucial, together with efforts to link efficacy to defined constituents and molecular targets rather than to provenance alone (Hanna et al., 2025).

Within this context, the considered *A. sinensis* (AS) extract was initially carefully analyzed for its phytochemical constituents. This analytical step is consistent with previous work on *A. sinensis,* emphasizing quality control through chemical profiling and the use of marker constituents (e.g., phthalides and phenolic acids) to support reproducibility and biological interpretation (Wei et al., 2016; Giacomelli et al., 2017). E/Z-ligustilide accounted for ∼1.2% (w/w) of the material. Furthermore, a series of isomer pairs of phthalic dimers that are known as Angesilides were detected but not quantified. Also, a series of phenolic derivatives of hydroxycinnamic acids, ferulic acid, and esters of caffeic acid with quinic acid have been identified, and their amount is 0.15%. Minor flavonoids (hesperidin and apigenin) were detected at ∼0.05% (w/w). In the extract, some coumarins, namely scopoletin, angelicin, psoralen, and phelloptorin, have been detected, but their amount was very limited and was not quantified.

In keratinocytes, AS significantly reduced LPS-induced NF-κB nuclear translocation, indicating a direct anti-inflammatory effect in the epidermal compartment. Keratinocytes are a valuable model for cutaneous inflammation due to their role as the first line of defense in the skin. They express pattern recognition receptors (PRRs) that detect pathogen-associated molecular patterns (PAMPs) and damage-associated molecular patterns (DAMPs), initiating the inflammatory cascade (Nestle et al., 2009). Our results are consistent with prior reports that suggest that the phenolic acids (e.g., ferulic acid) and phthalides can dampen inflammatory signaling in skin models (Działo et al., 2016; Liu et al., 2025). Previous studies on AS extracts have reported anti-inflammatory activity with down-modulation of NF-κB- and/or MAPK-associated readouts in experimental models (Lee et al., 2016; Lee et al., 2014; Chao et al., 2010)*.* In skin-relevant settings, topical AS has been reported to reduce inflammatory cytokines and NF-κB/MAPK activation in dermatitis-like models, which is consistent with the reduced p65 nuclear translocation observed in this study (Lee et al., 2016; Kim et al., 2018). Notably, this effect is within the range reported for other polyphenol-rich botanicals, which typically show a reduction in NF-κB activation at similar concentrations (Katiyar and Mukhtar, 2001).

UVB stimulation induced senescence in both HaCaT and BJ-5ta cells. Keratinocytes exhibit enhanced resistance to UVB-induced aging compared to dermal fibroblasts, primarily due to their superior DNA repair mechanisms and antioxidant defenses. The nucleotide excision repair (NER) pathway, crucial for repairing UVB-induced DNA damage, is more efficient in keratinocytes (Giglia-Mari and Sarasin, 2003). Additionally, keratinocytes express higher levels of antioxidant enzymes such as catalase and superoxide dismutase, providing better protection against reactive oxygen species generated by UVB exposure (Rhie et al., 2001). Across both HaCaT and BJ-5ta, AS showed an anti-senescent profile, with reduced SA-β-gal staining and lower *CDKN1A (p21)*. Although direct evidence for *A. sinensis* in cutaneous senescence models remains limited, ligustilide, one of the major *Angelica* phthalides, has been reported to modulate senescence-associated phenotypes and SASP-related outputs, providing a mechanistic context for the anti-senescent profile observed in this study (Wei et al., 2016; Takaya and Kishi, 2024). AS also lowered intracellular ROS in challenge conditions, supporting a link between redox control and the senescence readouts. While keratinocytes and fibroblasts display differential UVB sensitivity, our data indicate AS benefits both lineages. This pattern aligns with literature linking antioxidants to attenuation of stress-induced senescence markers and SASP output (Kim et al., 2019; Köllisch et al., 2005). The magnitude of this effect was consistent across replicate experiments within our experimental setting.

The effect of AS has also been tested on collagen production by fibroblasts. With aging and UV exposure, collagen production decreases while its degradation increases, leading to skin laxity and wrinkle formation (Fisher et al., 2009). By assessing the impact of AS on basal collagen production, we evidenced the ability of the extract to boost collagen levels even in the absence of an external stressor, suggesting support for extracellular-matrix maintenance. In line with this observation, prior studies have reported that *A. sinensis* preparations can modulate fibroblast repair-associated responses, including ECM-related outputs and oxidative-stress readouts (Hsiao et al., 2012). Differences in extraction chemistry, dosing, and assay design may explain variability across reports, particularly for migration-related endpoints. In addition, these results align with evidence that phenolics can restrain MMP activity and/or favour COL1A1 expression (Basu et al., 2019; Nichols and Katiyar, 2010). Mechanistically, this effect may be mediated through the TGF-β/Smad signaling pathway, a key regulator of collagen synthesis that has been shown to be modulated by other botanical polyphenols (Kang et al., 2003). Future studies should directly assess the impact of AS on this pathway. Interestingly, AS did not alter keratinocyte or fibroblast migration in our scratch assays under the conditions used, implying that benefits are more likely to arise from inflammatory control and ECM support than from effects on motility (Li et al., 2022; Yang et al., 2018; Ye et al., 2001). This selective action distinguishes AS from some other botanical extracts that have been shown to promote cell migration, potentially offering a more targeted approach to skin care (Pazyar et al., 2014).

The extract also displayed antibacterial and antifungal activity, with particularly strong effects against *Staphylococcus* species. Antimicrobial activity has also been reported for *Angelica* preparations *in vitro*, although potency varies substantially with extraction chemistry and formulation matrix, reinforcing the need to confirm performance in finished-product preservative challenge testing (Han and Guo, 2012; Wedge et al., 2009; Sowndhararajan et al., 2017). Elevated cutaneous microbial burden can contribute to inflammatory signaling and oxidative stress; therefore, antimicrobial activity observed *in vitro* may be consistent with reduced microbe-associated inflammatory pressure and support formulation robustness. However, effects on microbiome balance (i.e., dysbiosis) require dedicated community-level models and cannot be inferred from growth-inhibition assays alone (Kreouzi et al., 2025; Ni et al., 2022). Accordingly, these findings should be complemented by preservative challenge testing in finished products, where matrix effects, use levels, and packaging conditions ultimately determine real-world microbial protection.

In summary, this standardized AS extract (containing ∼1.2% total E/Z-ligustilide and a defined phenolic fraction) shows a coherent multimodal profile: it attenuates keratinocyte inflammatory signaling, reduces UVB-associated senescence readouts in both epidermal and dermal lineages, supports COL1A1-related signals in fibroblasts, and inhibits several skin-relevant microbes *in vitro*, without affecting migration under the conditions tested. These findings support further evaluation in advanced skin models and finished formulations before inferring topical performance.

## Data Availability

The raw data supporting the conclusions of this article will be made available by the authors, without undue reservation.

## Supplementary material

SUPPLEMENTARY FIGURE S1 LC-DAD trace at 254 nm with relevant UV spectra. LC-DAD chromatogram of Angelica sinensis (AS) extract recorded at 254 nm. Representative UV spectra of the main annotated peaks are shown (λ_max indicated). Peak annotations correspond to compounds reported in Tables 1,2.

SUPPLEMENTARY FIGURE S2 Senescence-associated β-galactosidase (SA-β-gal) staining and quantification in UVB-induced senescent keratinocytes and fibroblasts. Representative SA-β-gal staining images (10×) and quantification of SA-β-gal-positive cells in HaCaT keratinocytes and BJ-5ta fibroblasts. **(A,B)** Effect of UVB irradiation on the percentage of SA-β-gal-positive cells compared with non-irradiated control in HaCaT **(A)** and BJ-5ta **(B)**. **(C,D)** Effect of AS 30 μg/mL on the percentage of SA-β-gal-positive cells compared with untreated control in HaCaT **(C)** and BJ-5ta **(D)**. Data are presented as mean ± SD from n = 5 independent biological replicates. Statistical analysis: one-way ANOVA with Dunnett’s post hoc test vs. control; *****p* < 0.0001 UVB vs. basal control.
