# Supplementary material for: Activity of Angelica sinensis extract for cutaneous applications: antioxidant, anti-senescent, and antimicrobial effects
Source: Front Pharmacol. 2026 Mar 23;17:1779635. doi: 10.3389/fphar.2026.1779635 (PMC13050891; doi:10.3389/fphar.2026.1779635)
Supplement: Supplementary file 1 [file Table1.docx]

**Supplementary Tables**

**Table S1.** Gradient used for the Liquid Chromatography Diode Array multiple stage mass spectrometry (LC-DAD-MS^n^) experiments. Solvents were A water 1% formic acid, solvent B acetonitrile, solvent C methanol.

| **Time [min]** | **A [%]** | **B [%]** | **C [%]** | **Flow [mL/min]** | **Max Pressure Limit [bar]** |
| --- | --- | --- | --- | --- | --- |
| 0.00 | 95.0 | 0.0 | 5.0 | 0.400 | 400.00 |
| 15.00 | 90.0 | 5.0 | 5.0 | 0.400 | / |
| 30.00 | 65.0 | 10.0 | 25.0 | 0.400 | / |
| 35.00 | 65.0 | 10.0 | 25.0 | 0.400 | / |
| 45.00 | 30.0 | 20.0 | 50.0 | 0.400 | / |
| 55.00 | 0.0 | 40.0 | 60.0 | 0.400 | / |
| 60.00 | 0.0 | 70.0 | 30.0 | 0.400 | / |
| 65.00 | 95.0 | 0.0 | 5.0 | 0.400 | / |

**Table S2.** Gradient used for the Ultra Performance Liquid Chromatography Quadrupole Time of Flight Mass spectrometry (UPLC-QTOF) analysis. Solvents were A water 0.1% formic acid, solvent B acetonitrile, solvent C methanol.

| **Time [min]** | **A [%]** | **B [%]** | **C [%]** | **Flow [mL/min]** |
| --- | --- | --- | --- | --- |
| 0.00 | 95.0 | 5.0 | 0.0 | 0.300 |
| 2.00 | 95.0 | 5.0 | 0.0 | 0.300 |
| 14.50 | 10.0 | 15.0 | 75.0 | 0.300 |
| 15.00 | 10.0 | 15.0 | 75.0 | 0.300 |
| 16.50 | 0.0 | 25.0 | 75.0 | 0.300 |
| 17.00 | 0.0 | 25.0 | 75.0 | 0.300 |
| 18.00 | 95.0 | 5.0 | 0.0 | 0.300 |
