# Supplementary figures and images for: Activity of Angelica sinensis extract for cutaneous applications: antioxidant, anti-senescent, and antimicrobial effects
Source: Front Pharmacol. 2026 Mar 23;17:1779635. doi: 10.3389/fphar.2026.1779635 (PMC13050891; doi:10.3389/fphar.2026.1779635)

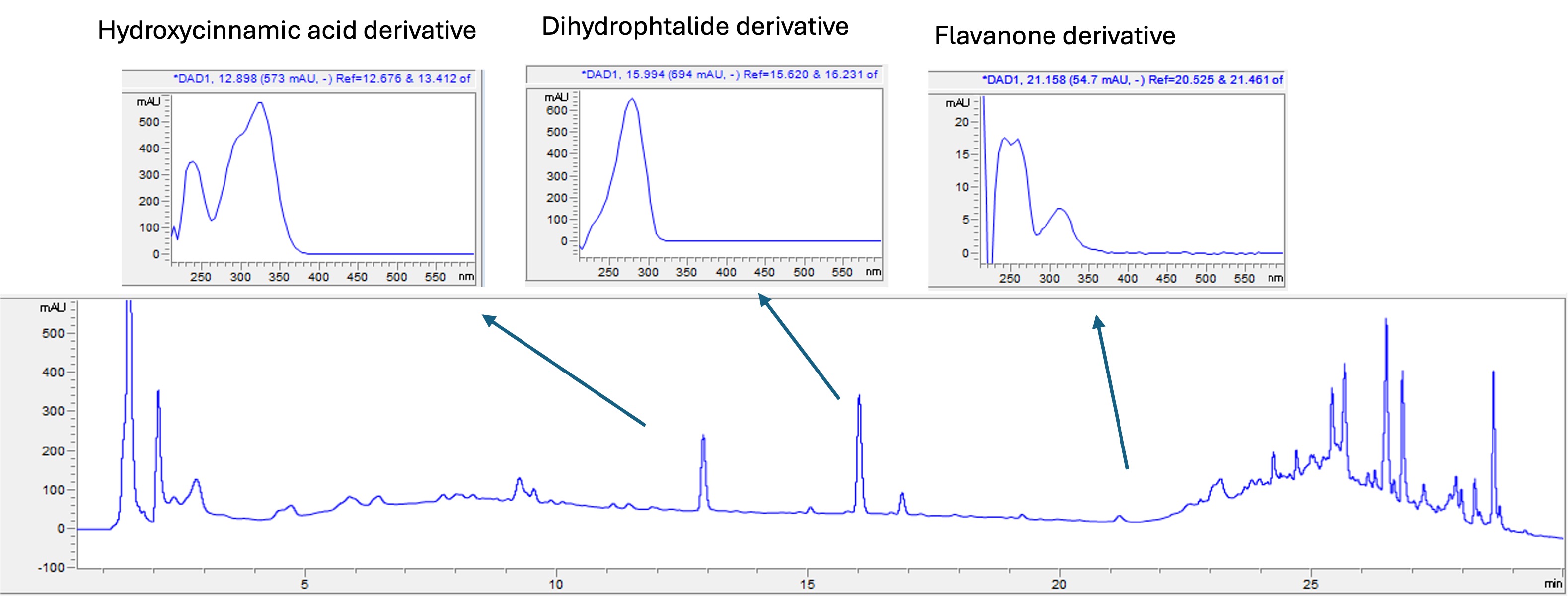

Supplement: Supplementary file 2 [file Image1.jpeg]

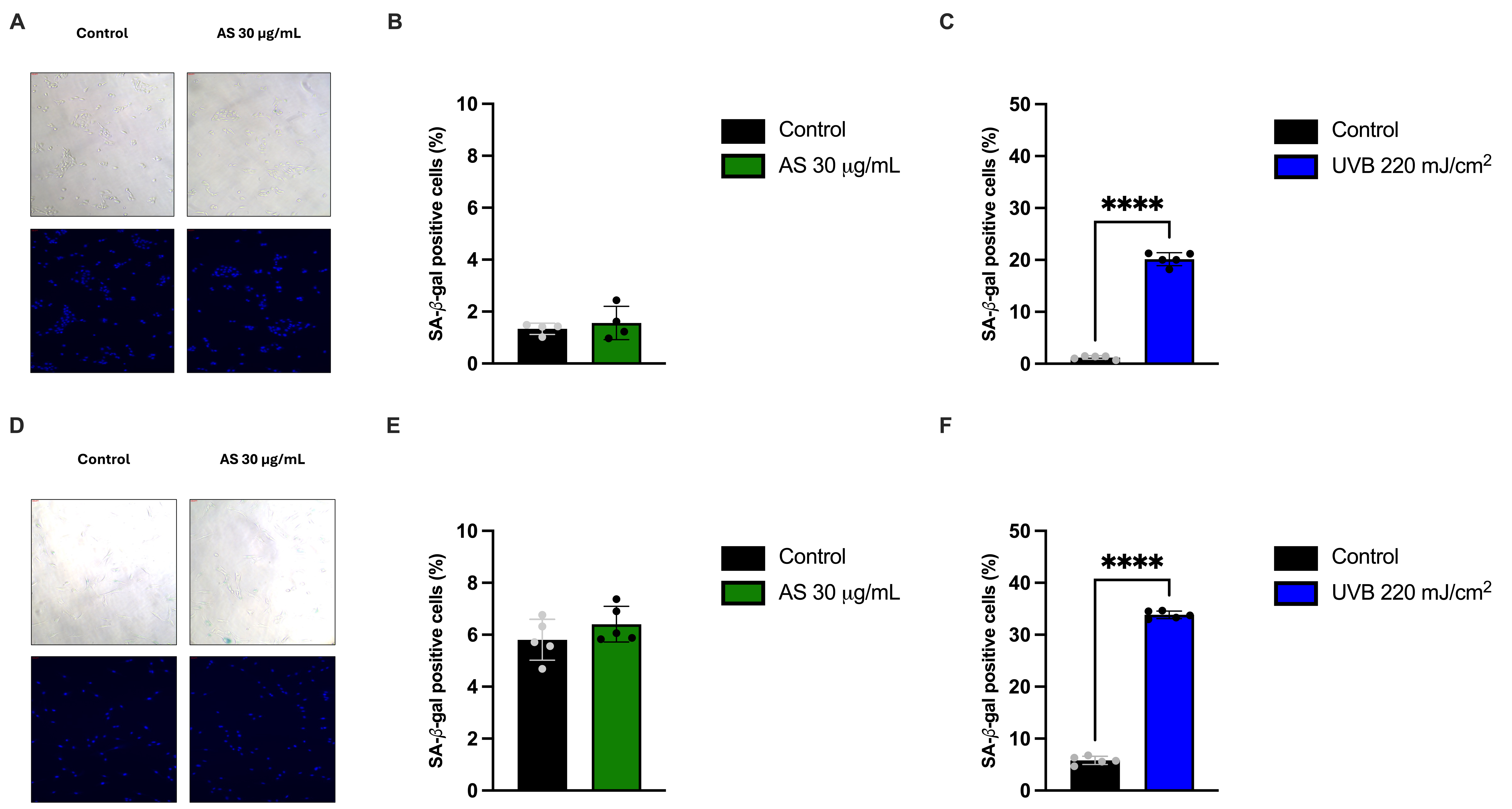

Supplement: Supplementary file 3 [file Image2.tiff]
